# Alcohol as a Medicine

**Published:** 1902-11-29

**Authors:** 


### Hospital Clinics and Medical Progress.

### ALCOHOL AS A MEDICINE.

However desirous medical men may be to stand aloof from the clamour of the wordy contest which rages perennially around the question of the use of alcohol as a beverage, all who use alcohol as a medicine are bound to arrive at definite and crisp conclusions in regard to its action when so given. Sir William Broadbent [1] has lately pointed out that whenever alcoholic stimulants are employed in the treatment of disease they ought to be regarded as medicines and prescribed with the same care and in the same definite way as the most powerful remedies. Before prescribing alcohol, whether in chronic or acute disease, we ought to ask ourselves the question, what we expect of them, and in what way the good effects we look for are to be produced. Moreover, we must bear in mind in regard to alcohol, as with other remedies, not only the direct and immediate advantage we are aiming at, but the collateral and remote effects which may be produced by it, especially if its use should be long continued.

As to the good we expect from the administration of alcohol, the idea so firmly fixed in the mind of the public that stimulants give strength must no longer be entertained for a moment. Such energy as is liberated by its oxidation can only be in the form of heat which is unimportant; it certainly does not give muscular force or nervous energy. The action of alcohol which we call stimulant is indirect. Its most conspicuous evidence is dilatation of the arterioles and capillaries which allows a freer supply of blood to all the organs. Thus the temporary general acceleration of the circulation, and the increased afflux of blood to the brain and viscera generally constitutes the action of alcohol of which we take advantage clinically. Such afflux may permit the evolution of functional energy, but this is provided at the expense of the blood and tissue, and is not supplied by the alcohol.

In prescribing alcohol in chronic disease the first thing is to take care not to do harm. Alcohol has no place in the treatment of weakness in childhood. In the anæmia and chlorosis of adolescence stimulants are of very doubtful advantage. But the most treacherous employment of stimulants at any period of life is their administration for the relief of depression, or of sensations described as "sinking," or of subjective feelings of weakness, even though such subjective sensations be accompanied by weakness of the pulse. Doubtless the immediate effect of the administration of alcohol under such circumstances is distinct and agreeable, but reaction is inevitable, and "if stimulants taken on medical advice are ever responsible for the establishment of the alcoholic habit, it is under these circumstances." Disease of either kidney or liver may almost be regarded as a bar to stimulants. A good word however is to be said, according to the experience of Dr. A. Ransome, for the use of alcohol in phthisis and tuberculous diseases. In debility, moreover, stimulants properly employed are of great value. They should only be taken with meals and their beneficial effects are to be estimated by the increase in the amount of food which is taken with their aid. In selecting a stimulant then, the criterion is not its chemical constitution but its effect on the appetite and digestion. Speaking of meals, however, it is to be noted that the biscuit and glass of port or sherry so often allowed in the course of the morning do not come within the definition of a meal, and that the practice is not a legitimate employment of stimulants.

In acute febrile disease stimulants are now generally given with judgment and in moderation. Time was, however, when the treatment of fever practically resolved itself into the administration of brandy, and this idea has not even yet been entirely uprooted from the public mind. Thus we are constantly called upon to withstand the entreaties of friends who imagine that the obvious weakness of the patient calls imperatively for stimulants. In acute febrile disease stimulants should in no case be given in the early stages, but should be withheld as long as possible. Even when the patient is an alcoholic and may be thought to have become dependent upon stimulants, the safer course is to withhold them until it is clear that they are necessary. The indication for their employment is not muscular prostration, and still less any sense of weakness of which the patient may complain. The pulse is the main guide, and we must judge by its frequency and low tension, with dicrotism and anacrotism, rather than by its apparent strength. The effects must be watched. If the stimulant is doing good the pulse becomes less frequent, steadier, better sustained and less dicrotic.

Another indication for the use of alcohol is dryness of the tongue, especially when there are sordes about the lips, teeth, and palate. When stimulants promote sleep and diminish restlessness and agitation they are doing good. On the other hand, should they cause excitement, or sleeplessness, or increase the frequency of the pulse, or set up gastric or intestinal derangement, they are doing harm. The odour of the breath is to some extent a guide. In febrile complaints the smell of wine or spirits very quickly disappears from the breath. If it linger or if the foul after-odour of the spirit drinker be recognised the stimulant should be withdrawn or the dose be diminished. It is very rarely necessary to give more than 6 ounces of brandy or whisky, or their equivalent in the form of wine, in enteric fever. In pneumonia stimulants may have to be pushed rapidly but 6 ounces to 8 ounces should be considered a very large amount, and 10 ounces a day represents a maximum in any form of acute disease. Effects can sometimes be obtained from champagne which cannot be got from spirits, and port or other wine may have a valuable place, especially perhaps during convalescence.

[1] Practitioner, November.

